# Influence of Excipients on the Release Kinetics and Antioxidant Activity of Encapsulated Propolis Phenolic Compounds

**DOI:** 10.3390/antiox15060767

**Published:** 2026-06-19

**Authors:** Monika Jokubaite, Mindaugas Marksa, Olexandr Nefodov, Tetiana Sakharova, Kristina Ramanauskiene

**Affiliations:** 1 Faculty of Pharmacy, Lithuanian University of Health Sciences, Sukileliai Avenue 13, LT-50162 Kaunas, Lithuania; monika.jokubaite@lsmu.lt (M.J.); mindaugas.marksa@lsmu.lt (M.M.); 2 Department of General and Clinical Pharmacy, Odesa I.I. Mechnikov National University, Elizavetinska 14, 65082 Odessa, Ukraine; o.nefodov@onu.edu.ua (O.N.); ssts2012.2010@gmail.com (T.S.)

**Keywords:** propolis extract, encapsulation, bioavailability, poloxamer 407, HPMC

## Abstract

Propolis is a widely studied natural raw material, the composition of which varies depending on the plant origin, harvest season, geographical area, climate and bee species. This large variety of chemical composition limits the use of propolis extracts in the pharmaceutical industry, which makes it difficult to ensure standardization of the raw material. One of the challenges that limit the modeling of oral pharmaceutical forms with propolis extract is the limited solubility and bioavailability of active compounds. Solid dispersion technology is commonly used in the production of oral capsules. The aim of this study is to evaluate the influence of different materials (HPMC, poloxamer and *β*-cyclodextrin) on the dissolution kinetics of phenolic compounds of propolis dry extract contained in capsules and their antioxidant activity in vitro. Analysis of the selected formulations showed that the major phenolic compounds detected in the propolis extract were also present in the dissolution medium samples. The auxiliary polymeric materials selected for the capsules formed a prerequisite for the dissolution kinetics profile. The addition of poloxamer and cyclodextrin increased the solubility and dissolution kinetics of hydrophobic propolis compounds in the test media. The addition of HPMC prolonged the dissolution kinetics of propolis active compounds. The antioxidant activity of the tested samples depends on the concentration of active compounds in the receptor medium by both the ABTS and DPPH methods.

## 1. Introduction

Propolis is a resinous mixture produced by bees, which bees collect from plants and use to protect their hive. Propolis is a widely studied raw material with a wide range of chemical compositions that vary depending on several parameters, such as plant origin, harvesting seasons, geography, type of bee flora, climatic changes and bee species at the collection site, and its biological properties are widely described in the scientific literature [1,2,3,4,5]. More than 300 different compounds have been isolated and identified from this natural product [6]. The main part of the propolis raw material consists of resins, rich in phenolic compounds, which determine the wide range of biological effects [7,8,9]. Numerous studies conducted on various propolis samples have shown that the main secondary metabolites are phenolic substances, especially flavonoids belonging to different subclasses, such as flavanones, flavones, flavonols and dihydroflavonols, which constitute more than 50% of the weight of propolis [7,10,11]. A wide matrix of chemical composition is directly associated with the biological effects of propolis. Propolis raw material is used to strengthen the immune system, treat colds due to its antibacterial and antiviral effects, as a remedy for skin problems, to treat small ulcers and aphthae in the oral cavity or finally restore the balance of the gastric mucosa [1,5,6,11,12,13]. The antioxidant activity of propolis mainly depends on the flavonoid and polyphenol fraction [14]. The high antioxidant activity of these compounds is associated with their ability to donate hydrogen atoms and electrons of the aromatic hydroxyl group to free radicals and the possibility of charge delocalization in the double-bond system of the aromatic ring [15]. The commercial use of propolis in pharmaceutical products is most limited by the large variation in chemical composition, which is determined by the diversity of botanical origin, variability of plant precursors, seasonality and climatic conditions, which makes it difficult to ensure the standardization and traceability of the raw material [16]. Dry propolis extracts obtained by controlled extraction and drying technologies (vacuum or spray drying) allow the stabilizing of phenolic compounds and ensure dosing accuracy [17]. By encapsulating the dry extract of propolis, it is possible to modulate the release of active ingredients in a targeted manner, both by protecting them from degradation in the digestive tract and by controlling diffusion mechanisms, which is relevant when developing oral systems with modified or local biological effects. One of the problems that must be addressed when modeling oral pharmaceutical forms with propolis extract is the limited solubility of active compounds [18]. Propolis resins are hydrophobic and include highly valuable compounds, such as flavonoids and phenolic acids, which have are low polarity and are characterized by poor solubility in water. Scientific research data reveal that propolis active substances are extremely poorly soluble in aqueous media; the resinous structure and wax components block the effective release of phenolic compounds into water, which limits their use in pharmaceuticals [13,19,20]. Propolis active compounds (phenolic compounds) are characterized by limited oral bioavailability, which is mainly determined by their poor solubility in aqueous media. It can be stated that poor solubility in water together with intensive metabolism in the digestive tract are the main barriers determining poor bioavailability [21,22,23,24,25]. To overcome these obstacles, scientists propose the use of cyclodextrins, which increase solubility and protect the active components of propolis from biotransformation in the body [26]. Solid dispersion (SD) technology is often used in the production of capsules. Scientific studies confirm that poloxamers are suitable for increasing the bioavailability of propolis. Their use allows for the successful transformation of hydrophobic propolis extract into a well-absorbed oral form (capsules), ensuring a more effective therapeutic effect. Poloxamer acts as a carrier that not only physically disperses hydrophobic particles of poor solubility, but also reduces their crystallinity, turning them into an amorphous form. This allows a faster release of active ingredients and their penetration through intestinal membranes [27]. Cyclodextrins can also be used as excipients in capsule formulations to improve the solubility and dissolution rate of poorly water-soluble compounds. By forming complexes, hydrophobic components are partially encapsulated in the cyclodextrin cavity, while the hydrophilic outer layer facilitates dispersion in aqueous gastrointestinal fluids. This reduces aggregation and increases the dissolution rate [28,29,30]. Extended release is another way to more effectively absorb poorly soluble active ingredients. HPMC (hydroxypropyl methylcellulose) is a widely used hydrophilic matrix polymer, which, due to its ability to form a viscous gel barrier, ensures a controlled and prolonged release of active ingredients [31,32,33]. The aim of this study is to evaluate and compare the influence of different excipients (HPMC, poloxamer and β-cyclodextrin) on the dissolution kinetics of encapsulated propolis dry extract phenolic compounds and their antioxidant activity in vitro.

## 2. Materials and Methods

### 2.1. Materials

All reagents, standards, and solvents were used at analytical grade. Caffeic acid (≥98%, HPLC) was purchased from Sigma-Aldrich Chemie GmbH (Steinheim, Germany). D(+)-glucose monohydrate, starch, hypromellose (HPMC, USP testing specifications), poloxamer 407 (Kolliphor P 407, Ph. Eur./USP/NF pharmaceutical grade, containing 71.5–74.9% oxyethylene), *β*-cyclodextrin (≥97% purity, bio-reagent grade), and PROSOLV SMCC 50 were obtained from Sigma-Aldrich GmbH (Buchs, SG, Switzerland), respectively, and all were used as excipients. Ethanol (96%) was purchased from AB “Vilniaus degtine” (Vilnius, Lithuania). Phosphate-buffered saline (PBS) (pH 7.4) was obtained from Gibco (Paisley, UK). Ultrapure water was produced using a water purification system Milli-Q (Millipore, Arlington, MA, USA). Chromatographic grade acetonitrile and trifluoroacetic acid (TFA, ≥99.0% purity) were obtained from Sigma-Aldrich Chemie GmbH (Steinheim, Germany). Crude propolis was harvested during the autumn of 2024 from Raseiniai region, Lithuania.

### 2.2. Preparation of Propolis Extract

The propolis extract was prepared using a combined maceration- and ultrasound-assisted extraction method. An amount of 20 g of crushed propolis raw material was poured into 100 mL of 70% (*v*/*v*) ethanol solution. The mixture was shaken in a laboratory shaker at 400 rpm at a temperature of 40 °C for 2 h. Subsequently, the extraction mixture was macerated for 5 days in the dark, shaken once daily for an additional 1 h. After, the propolis extract was extracted in an ultrasonic bath (Sonic 10, 40 kHz) for 15 min, ensuring that the final extraction temperature did not exceed 42 °C. After ultrasonic extraction, the extract obtained was filtered through filter paper. The propolis extract was mixed with silica, MCC (microcrystalline cellulose) carrier in a ratio of 20:3:7 and lyophilized using a freeze dryer (LyoQuest Telstar, Terrassa, Spain) to obtain a dry mass, which was ground to powder. The prepared dry extract was stored in a tightly closed container until further studies.

The total phenolic content of the dry extract was determined. A fixed amount of the extract (100 mg) was dissolved in 70% (*v*/*v*) ethanol using a magnetic stirrer (IKAMAG C-MAG HS7, IKA-Werke GmbH & Co. KG, Staufen, Germany) at 1250 rpm for 30 min. To remove undissolved particles, the mixture was centrifuged and subsequently filtered through a (0.45 µm) membrane filter. The total phenolic content in the filtrate was analyzed spectrophotometrically using the Folin–Ciocalteu method, as described in Section 2.6.

### 2.3. Encapsulation Process

For encapsulation of the propolis extract, hard gelatin capsules (size No. 0) were used. The powder mixture for encapsulation was prepared by mixing dry propolis extract with selected combinations of excipients. The compositions of the encapsulated mixtures are presented in Table 1. The capsules were manufactured using a capsule-filling machine (Capsuline, Davie, FL, USA). Each capsule was dosed with 200 mg of dry propolis extract. The manufactured capsules were divided into three formulation groups according to the excipients used. In the first group, PROSOLV SMCC was used as a filler together with poloxamer 407. In the second group, PROSOLV SMCC and *β*-cyclodextrin were used. In the third group, PROSOLV SMCC and hypromellose (HPMC) were used. Poloxamer 407 and *β*-cyclodextrin were used as solubility-enhancing components in the capsule formulations, and HPMC was selected as an excipient modifying the dissolution kinetics. The dry extract was analyzed and showed a total phenolic content of 233.77 ± 18.05 mg/g (expressed as *p*-coumaric acid equivalents).

The amounts of the excipients were selected based on our previous research experience with caffeic acid and *p*-coumaric acid capsule formulations [34,35]. The weight of the excipient and extract mixture was 300 mg per capsule (2:1 ratio of extract to total excipient by weight).

### 2.4. Disintegration Test

The disintegration time of the capsules was determined using a modified method according to the European Pharmacopoeia method (Ph. Eur. 2.9.1) [36]. The test was performed using an IKAMAG C-MAG HS7 (IKA-Werke GmbH & Co. KG, Staufen im Breisgau, Germany) magnetic stirrer. A 0.1 M hydrochloric acid solution was used as the disintegration medium. The temperature of the medium was maintained at 37 ± 0.5 °C during the test.

### 2.5. Dissolution Test 

The capsule dissolution test was performed according to the European Pharmacopoeia method (Ph. Eur. 2.9.3) [36] using a paddle-type dissolution apparatus Sotax AT 7smart (SOTAX AG, Allschwil, Switzerland) with a sinker. Two dissolution media were used for the dissolution test: 0.1M HCl (pH 1.2) and phosphate-buffered solution (pH 6.8) and. The first medium (pH 1.2) was used to simulate the acidic environment of the stomach. The second medium (pH 6.8) was used to simulate the physiological conditions of the small intestine. The volume of the dissolution medium was 500 mL, and the temperature was maintained at 37.0 ± 0.5 °C. The stirring speed was set at 100 rpm. Samples of the dissolution medium were taken after 5, 10, 15, 30, 45, 60 and 90 min, each time taking a sample of a fixed volume and replacing it with the same amount of fresh medium. The collected dissolution medium samples were analyzed using the UV spectrophotometric Folin–Ciocalteu method described in Section 2.6.

Release kinetics were evaluated using zero-order, Higuchi, and Korsmeyer–Peppas kinetic models, which are widely applied to characterize dissolution and matrix-controlled release profiles [37,38].

### 2.6. Determination of Total Phenolic Content

The total phenolic content of the propolis extract was determined using the Folin–Ciocalteu spectrophotometric method according to Singleton et al. with some modifications [39]. For analysis, 1.0 mL of the test extract solution was transferred to a 25 mL volumetric flask. 5.0 mL of 0.2 N Folin–Ciocalteu reagent was added. After 5 min of incubation, 4.0 mL of 7.5% sodium carbonate solution was added. The reaction mixture was diluted with distilled water to a final volume of 25 mL and mixed well. The prepared solution was incubated for 30 min at room temperature in the dark. After incubation, the absorbance was measured with an Agilent 8453 spectrophotometer (Agilent Technologies, Inc., Santa Clara, CA, USA) at a wavelength of 765 nm. The total phenolic content was expressed as mg *p*-coumaric acid equivalents (CAE). All measurements were performed in triplicate.

### 2.7. Phenolic Compounds Determination by HPLC

The phenolic compounds of propolis and their content in the tested samples were determined using a previously validated high-performance liquid chromatography (HPLC) method [40,41]. Qualitative and quantitative analysis was performed using a Waters 2695 Alliance chromatography system (Waters, Milford, MA, USA) equipped with a Waters 996 photodiode array detector. The resulting data were processed with the Empower 2 Chromatography Data Software (Waters Corporation, Milford, MA, USA). Chromatographic separation was performed using an ACE column (C18, 250 mm × 4.6 mm, particle size 5 µm). The mobile phase consisted of eluent A (0.1% TFA) and eluent B (acetonitrile). Gradient elution was performed according to the following program: 0–8 min, 5–15% B; 8–30 min, 15–20% B; 30–48 min, 20–40% B; 48–58 min, 40–50% B; 58–65 min, 50% B; 65–66 min, 50–95% B; 66–70 min, 95% B; and 70–71 min, 95–5% B. The mobile phase flow rate was 1 mL/min, the injection volume was 10 µL. The column temperature was maintained at 25 °C. Reference compounds included *p*-coumaric acid (RT = 19.67 min), cinnamic acid (RT = 43.25 min), caffeic acid (RT = 14.09 min), pinocembrin (RT = 57.97 min), vanillin (RT = 17.49 min), vanillic acid (RT = 13.46 min), ferulic acid (RT = 21.89 min). The linearity of calibration curves for the reference compounds ranged from R^2^ = 0.9998 to 0.9999.

### 2.8. Determination of Antioxidant Activity by DPPH and ABTS Radical Scavenging Assay

The antioxidant activity of the propolis extract was assessed using DPPH (2,2-diphenyl-1-picrylhydrazyl) and ABTS (2,2′-azino-bis (3-ethylbenzothiazoline-6-sulfonic acid)) radical scavenging assays. The ABTS•+ radical scavenging assay was performed according to the method described by Re et al. with certain modifications [42]. For analysis, 3 mL of ABTS•+ working solution (abs 0.800 ± 0.02, 734) was mixed with 10 μL of the test sample (media after dissolution test for 60 min). The reaction mixtures were kept in the dark at room temperature for 30 min. The DPPH free radical scavenging activity was determined according to the method described by Brand-Williams et al. with certain modifications [43]. For analysis, 3 mL of DPPH solution (0.1 mM) in 96.3% (*v*/*v*) ethanol was mixed with 10 μL of the test sample (medium after dissolution test for 60 min). The reaction mixtures were incubated in the dark at room temperature for 30 min. Absorbance was measured at a wavelength of 517 nm. The results of both reactions were evaluated with an Agilent 8453 spectrophotometer (Agilent Technologies, Inc., Santa Clara, CA, USA).

Radical scavenging activity was calculated as a percentage inhibition according to the formula [44]:

Radical scavenging activity (%) = [(A_0_ − A_s_)/A_0_] × 100

where A_0_ is the absorbance of the control solution, and A_s_ is the absorbance of the test sample. All measurements were performed in triplicate, and the results are presented as the mean ± standard deviation.

### 2.9. Statistical Analysis

Statistical analysis was performed using GraphPad Prism (Version 11.0.1, San Diego, CA, USA) and Microsoft Excel 2019 (Microsoft Corporation, Redmond, WA, USA). Three capsules were analyzed for each formulation. Results are presented as mean ± standard deviation (SD) (*n* = 3). For disintegration time evaluation, statistical differences between formulations were assessed using one-way ANOVA followed by Dunnett’s T3 multiple comparisons test. Release profiles in PBS and HCl media were analyzed using two-way ANOVA to evaluate the effects of formulation type, sampling time, and their interaction, followed by Tukey’s post hoc multiple comparisons test. Antioxidant activity data obtained from dissolution medium samples were analyzed separately for DPPH and ABTS assays using one-way ANOVA followed by Dunnett’s multiple comparisons test against the control formulation (C0). Correlations between phenolic compound release and antioxidant activity were evaluated using Spearman rank correlation. Differences were considered statistically significant at *p* < 0.05.

## 3. Results and Discussion

### 3.1. Disintegration of Encapsulated Propolis Dry Extract Formulations

**Figure 1:**
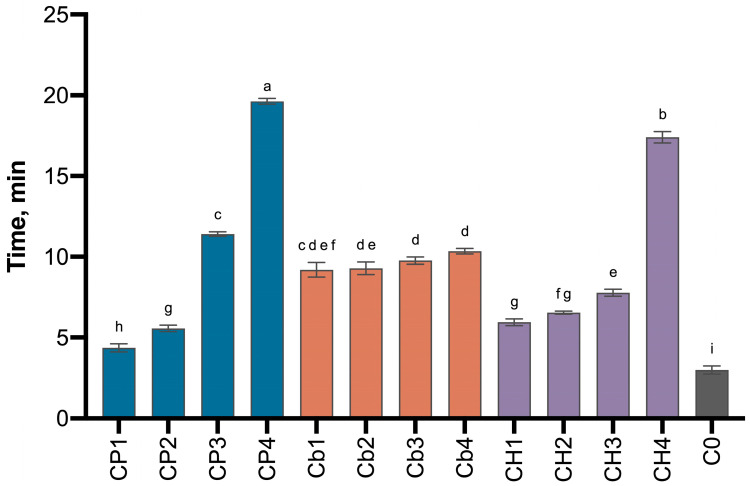
Disintegration time (min) of capsules containing propolis extract formulated with different excipients compared with the control formulation. Values are expressed as mean ± SD (*n* = 3). Different letters indicate statistically significant differences between formulations according to one-way ANOVA followed by Dunnett’s T3 multiple comparisons test (*p* < 0.05).

A disintegration study of the manufactured capsules was conducted. This test is considered a critical quality control step, the results of which are an informative tool to ensure that the solid dosage form physically disintegrates into small particles within a specified time [45]. This directly accelerates the subsequent dissolution kinetics. The results of the study are presented in Figure 1.

The disintegration study showed that all tested capsule formulations disintegrated within 30 min, indicating that the modeled capsules met the general disintegration requirements for hard capsules. The control formulation (C0) was characterized by the shortest disintegration time, the capsule shell and propolis dry extract did not significantly slow down the penetration of the medium into the capsule content. In the CP1–CP4 capsule group, a concentration-dependent increase in disintegration time was observed with increasing poloxamer 407 content. A statistically significant increase in disintegration time was observed in formulations containing a higher amount of P407. Such disintegration results may be related to the hydration behavior of poloxamer 407 and the formation of more viscous micellar or gel-like hydrated structures, which may limit the penetration of water into the capsule interior and delay the physical disintegration of the powder mass [31,46]. Capsules containing Cb1–Cb4 *β*-cyclodextrin exhibited similar disintegration times. This suggests that increasing the amount of *β*-cyclodextrin did not significantly prolong capsule disintegration, as observed for poloxamer 407. This behavior may be related to the physicochemical properties of native *β*-cyclodextrin, including its crystalline structure and limited solubility in water, which reduce its tendency to form a highly viscous hydrated matrix. Therefore, *β*-cyclodextrin may act primarily as a solid excipient modifying the powder structure and local wetting, rather than as a swelling or gel-forming component [47,48]. Capsules containing CH1–CH4 HPMC showed a gradual increase in disintegration time at lower polymer concentrations, while the formulation containing the highest amount of HPMC (CH4) showed a significant delay. The results of the study are related to the known hydration and swelling properties of HPMC, which can form a viscous, gel-like layer around the capsule contents. At higher polymer concentrations, such a hydrated barrier can limit the ingress of the medium and slow down the disintegration process [31,32]. The disintegration results show that the selected excipients significantly affected the physical disintegration of the capsules. Poloxamer 407 and HPMC showed a more pronounced slowing effect at higher concentrations, while *β*-cyclodextrin gave a more stable disintegration profile with less concentration-dependent variability. These results are important because capsule disintegration determines the initial contact of the powder contained in the capsule with the dissolution medium and may affect the subsequent release kinetics of propolis phenolic compounds [49].

### 3.2. Dissolution of Encapsulated Propolis Dry Extract Formulations

**Figure 2:**
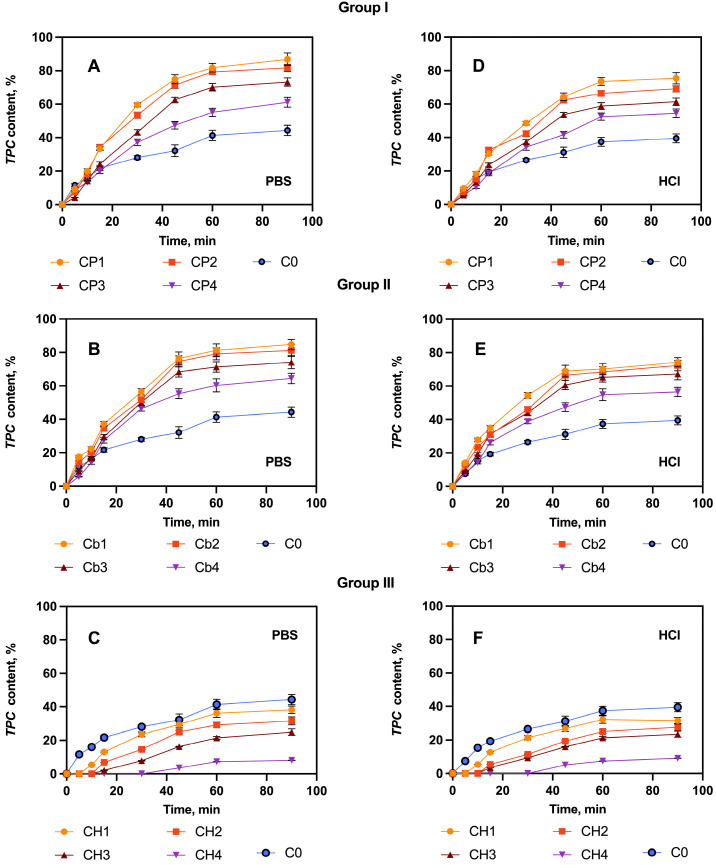
Release profile of total phenolic compounds from propolis extract capsules formulated with different excipients in PBS (**A**–**C**) and HCl medium (**D**–**F**). Group I (CP formulations), group II (Cb formulations), and group III (CH formulations) are compared with the control (C0). Results presented as mean ± SD (*n* = 3). Statistical significance between formulations and time points evaluated using ordinary two-way ANOVA followed by Tukey’s post hoc test (*p* < 0.05).

Dissolution kinetics of the encapsulated propolis extract was performed. The results of the study are presented in Figure 2. The results of the dissolution kinetics studies show the dominant influence of the carrier on the release of the active compounds of encapsulated propolis. The dissolution profiles of propolis active compounds from capsule preparations (groups I–III) were evaluated in two different media, namely PBS (pH 7.4) and HCl (pH 1.2).

Capsules of group I and group II were characterized by more efficient dissolution kinetics compared to capsules of group III. During the dissolution process from 15 to 60 min, the release rate of active substances depended on the auxiliary components of the encapsulated powder mixture and their respective concentrations. The pH value of the dissolution medium had a significant influence on the release efficiency of the active compounds of encapsulated propolis. A statistically significant difference (*p* < 0.05) was observed between the dissolved amount of the active compounds at the endpoint in PBS (pH 7.4) and HCl (pH 1.2) media for group I and II capsule formulations. The acceptor medium fills the pores of the matrix and the diffusion intensity depends on the solubility of the active substances and the speed of movement of molecules outward due to the high concentration gradient [50].

The in vitro release study of capsules in group I showed that the amount of poloxamer 407 in the capsules had a direct effect on the diffusion rate of the active compounds. All tested formulations (CP1–CP4) were characterized by significantly higher substance release compared to the control group (C0) (*p* < 0.05). In terms of the effect on release rate with increasing poloxamer content (from CP1 to CP4), the percentage of active compounds released from the propolis extract consistently decreased. CP1 (86.78 ± 3.80%) and CP2 (81.74 ± 2.10%) capsules released a statistically significantly higher amount of active compounds compared to the CP3 (61.16 ± 2.95%) and CP4 (44.30 ± 3.01%) capsules (*p* < 0.05). According to the European Pharmacopoeia (Ph. Eur. Chapter 5.17.1), a requirement for a typical immediate-release oral solid dosage form is that at least 75% of the active substance should be dissolved within 45 min under specified test conditions [51]. Therefore, the low cumulative release observed for the CP4 formulation showed a slower dissolution profile compared with rapidly releasing formulations. However, this extended-release profile demonstrates that the high amount of P407 (75 mg) resulted in a more prolonged release profile. It is likely that higher poloxamer concentrations (CP3 and CP4 groups) may be associated with the formation of a more viscous hydrated layer, which slowed down the diffusion of the active substance, although the overall solubility remained higher than that of the control sample [52]. When the capsule enters the dissolution medium, the poloxamer rapidly hydrates. At low concentrations (CP1), it may act as a surfactant; it improves the wetting of propolis extract particles and solubilizes them. Hydrophobic active compounds of propolis are incorporated into the micelle cores, thereby improving their solubility. It should be noted that at high concentrations of P407, a denser micellar network may form, which limits the movement of active compounds from the micelles to the dissolution medium. Poloxamer 407 is characterized by the ability to form thermoreversible gels. It can be assumed that the formation of an in situ gel barrier is one of the reasons for the slowdown in dissolution kinetics [53,54,55]. Such a hydrated barrier may limit water penetration and diffusion; water penetrates more difficultly, and dissolved propolis compounds must slowly diffuse through this viscous barrier to enter the environment.

From the capsules of group II, a significantly higher amount of active compounds was released in the buffered environment compared to the acidic environment. Cb1 and Cb2 had the fastest dissolution kinetics of all the capsules tested in this group. Capsules of the Cb4 composition are slow-dissolving capsules; the final dissolved amount reaches only about 64%. The Cb4 capsules contain the highest amount of *β*-cyclodextrin compared to other capsules in this group and are characterized by the slowest dissolution kinetics profile. Cyclodextrins are usually used to increase solubility; their excessive amount in solid pharmaceutical forms can cause the opposite effect. *β*-cyclodextrin has a crystalline structure, is poorly soluble in water, and its excessive amount changes the capillarity of the powder mixture, increases the density of the encapsulated mixture and acts as a physical barrier preventing the dissolution medium from penetrating the solid matrix [56]. The excess of cyclodextrin molecules forms a dense molecular lattice; a viscous diffusion layer is formed around the particles of the encapsulated mixture, thereby slowing down the diffusion of molecules from the encapsulated mixture into the dissolution medium [57,58]. The appropriate amount of cyclodextrin improves the dissolution kinetics of the active components of propolis. A statistically significantly higher (*p* < 0.05) amount of active compounds was released from the compositions Cb1 and Cb2 compared to the control and other formulations of this group. Cyclodextrins can improve the solubility of poorly water-soluble compounds by forming non-covalent inclusion complexes in which the lipophilic moiety is partially incorporated into a hydrophobic cavity, while the hydrophilic outer surface of the cyclodextrin improves the water compatibility of the complex [59].

The study data show that increasing HPMC concentration in group III capsules (from CH1 to CH4) proportionally slowed down the release kinetics of the tested phenolic compounds of propolis and increased the initial lag time. Compared to the control sample (C0), the HPMC additive formed a prolonged release system, in which the highest polymer concentration (CH4) limited the diffusion of active substances in the initial stages of the study. In PBS and acidic medium the release of active compounds varied from 8.16 ± 0.12% to 38.11 ± 2.30%. HPMC, as a hydrophilic polymer, forms a hydrogel barrier in contact with the dissolution medium [60]. As the polymer content increases, a denser and less permeable matrix is formed; therefore, the active compounds have to overcome a longer diffusion path [61]. It is hypothesized that intermolecular hydrogen bonds may form between the free hydroxyl groups of the HPMC polymer backbone and the phenolic compounds of the propolis extract. Such theoretical interactions could contribute to the stabilization of the hydrogel matrix network and modulation of the diffusion rate, but more detailed spectroscopic studies, such as FTIR analysis, are needed to formally confirm this mechanism.

The dissolution kinetics profiles were better fitted by the Higuchi and Korsmeyer–Peppas models than by the zero-order model, as indicated by the higher R^2^ values obtained for most of the formulations (Table 2). The results indicate that the release of phenolic compounds from the studied systems was mainly driven by diffusion-related processes rather than by a steady zero-order release kinetics. The CP formulations exhibited higher Korsmeyer–Peppas correlation coefficients together with n values greater than 1, suggesting polymer relaxation and swelling contributions to the release process. Most of the Cb formulations exhibited n values characteristic of anomalous transport behavior. The Korsmeyer–Peppas model was not reliably applied to the CH formulations due to the insufficient number of valid early release data points in the Mt/M∞ ≤ 0.60 region, which was associated with delayed and limited initial release in these systems.

**Figure 3:**
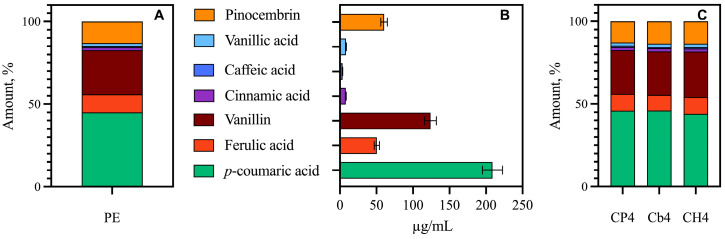
Phenolic compounds profile in propolis extract and capsules. (**A**)—propolis extract (PE) percentage contribution of individual phenolic compounds in the extract, (**B**)—quantitative content of the identified compounds expressed as µg/mL in the propolis extract, (**C**)—propolis percentage contribution of individual phenolic compounds in capsules CP4, Cb4, and CH4.

To fully assess how the maximum carrier loading affects the release kinetics of individual components, the formulations showing the slowest dissolution profile were purposefully selected for the analysis of the percentage distribution of phenolic compounds (CP4, Cb4 and CH4). The same major identified phenolic compounds were detected in the dissolution medium samples as in the propolis extract used for encapsulation. The results of the study demonstrate the predominance of *p*-coumaric acid, which accounts for the majority of all released phenolic compounds (around 45%) (Figure 3). Vanillin was also detected among the major identified phenolic compounds of the analyzed propolis extract, which accounts for about 26% of the total composition. Phenolic compounds in the propolis extract are important due to their antioxidant properties [62,63]. The obtained results encourage the pursuit of a more detailed analysis of the composition of active components using approved and validated analytical methodologies and thus contribute to the standardization of propolis products.

### 3.3. Antioxidant Activity

**Figure 4:**
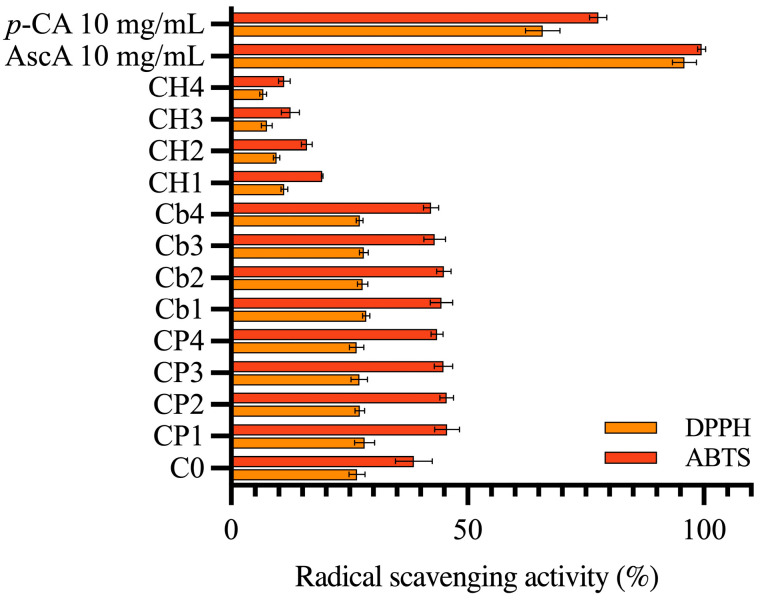
Antioxidant activity of propolis extract capsule formulation dissolution test solutions after 60 min evaluated by DPPH and ABTS radical scavenging assays, compared with *p*-coumaric acid 10 mg/mL (*p*-CA) and Ascorbic acid 10 mg/mL (AscA). Results expressed as radical scavenging activity (%), mean ± SD (*n* = 3).

To assess the influence of excipients on the stability of propolis phenolic compounds and their ability in acceptor medium to neutralize free radicals, antioxidant activity studies were performed using the DPPH and ABTS methods (Figure 4).

The experimental data showed that the antioxidant activity of all tested samples, determined by the ABTS method, was higher compared to the DPPH method. Phenolic compounds in extracts may differ in the number, position, conjugation, and oxidation-reduction potential of hydroxyl groups, so the same release of active compounds does not necessarily give the same DPPH or ABTS response. This behavior may be explained by structure–activity relationships characteristic of phenolic compounds [64,65], as well as the higher sensitivity of the ABTS radical and better solubility in both aqueous and organic media, which allows for a more accurate assessment of the reactivity of hydrophobic propolis components. When analyzing the control samples, it was found that ascorbic acid (10 mg/mL) and *p*-coumaric acid (10 mg/mL) were characterized by the highest free radical binding capacity.

**Figure 5:**
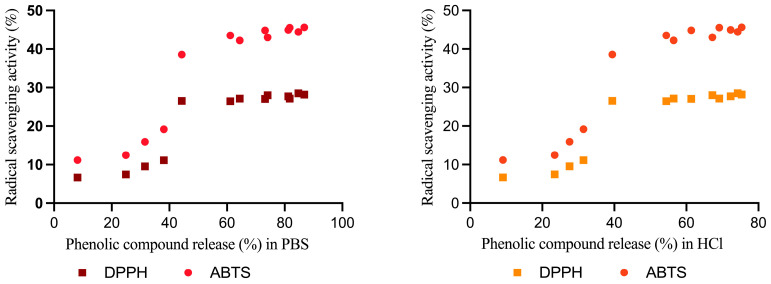
Correlation between phenolic compound release (%) and radical scavenging activity (%) determined by ABTS and DPPH assays in PBS and HCl dissolution media after 60 min release testing. Correlations were evaluated using Spearman rank correlation analysis.

The groups of test capsules showed a different antioxidant profile, directly dependent on the excipients used. Correlation analysis demonstrated strong associations between phenolic compound release and antioxidant activity of the dissolution medium samples (Figure 5). Strong positive correlations were observed between the phenolic compounds release of phenolic compounds and antioxidant activity in both dissolution media. In PBS medium, Spearman correlation coefficients reached r = 0.9341 for the ABTS assay and r = 0.9615 for the DPPH assay (*p* < 0.0001). In HCl medium, strong positive correlations were obtained for both ABTS (r = 0.9286, *p* < 0.0001) and DPPH assays (r = 0.9670, *p* < 0.0001). These results indicate that increased phenolic compound release was closely associated with enhanced radical scavenging activity regardless of the dissolution medium. The results of the antioxidant activity study pose challenges for more detailed studies to assess the contribution of individual propolis active components to the antioxidant activity of the formulations.

## 4. Conclusions

The results of the study show that the selected polymeric excipients had an impact on the solubility properties and antioxidant activity of propolis active compounds in vitro. Disintegration and dissolution studies are important quality control tools for oral dosage forms. Carriers in capsules that increase solubility (poloxamer, cyclodextrins) were associated with faster and greater neutralization of free radicals. Moderate-to-strong correlations were observed between phenolic compound release and antioxidant activity in both PBS and HCl media. The results show that the studied polymer systems modify the release of active compounds from propolis extract, and for a deeper understanding of the interactions and mechanisms involved, it is necessary to conduct more detailed studies of the physicochemical properties of the materials using modern analytical methods. In order to achieve reliable standardization and practical application of propolis capsules, it is appropriate to conduct a broader analysis of the chemical composition using validated analytical methods.

## Figures and Tables

**Table 1:** Composition of propolis capsule formulations with different excipient systems (amount per capsule, mg).

		Propolis	P407	Prosolv	*β*-Cyclodextrins	HPMC	Total Mass
	C0	200					200
Group I	CP1	200	10	90			300
	CP2	200	25	75			
	CP3	200	50	50			
	CP4	200	75	25			
Group II	Cb1	200		90	10		
	Cb2	200		75	25		
	Cb3	200		50	50		
	Cb4	200		25	75		
Group III	CH1	200		90		10	
	CH2	200		75		25	
	CH3	200		50		50	
	CH4	200		25		75	

**Table 2:** Release kinetic parameters of propolis capsule formulations in PBS and HCl media obtained using zero-order, Higuchi, and Korsmeyer–Peppas models.

	Formulation	Zero-Order R^2^	Higuchi R^2^	Korsmeyer–Peppas R^2^	n
PBS	CP1	0.858	0.950	0.998	1.170
	CP2	0.969	0.947	0.998	1.365
	CP3	0.883	0.943	0.994	1.620
	CP4	0.948	0.972	0.924	0.887
	Cb1	0.847	0.959	0.880	0.648
	Cb2	0.937	0.951	0.924	0.766
	Cb3	0.843	0.941	0.984	1.034
	Cb4	0.840	0.942	0.997	1.434
	CH1	0.886	0.935	N/A	N/A
	CH2	0.414	0.899	N/A	N/A
	CH3	0.951	0.878	N/A	N/A
	CH4	0.882	0.731	N/A	N/A
	C0	0.869	0.986	0.983	0.559
HCl	CP1	0.867	0.957	0.993	1.046
	CP2	0.810	0.942	0.973	1.313
	CP3	0.896	0.949	1.000	1.318
	CP4	0.883	0.956	0.966	1.261
	Cb1	0.808	0.954	0.984	0.839
	Cb2	0.847	0.960	0.996	0.807
	Cb3	0.840	0.954	0.999	1.091
	Cb4	0.644	0.957	0.979	1.081
	CH1	0.396	0.918	N/A	N/A
	CH2	0.935	0.902	N/A	N/A
	CH3	0.940	0.895	N/A	N/A
	CH4	0.896	0.754	N/A	N/A
	C0	0.848	0.977	0.886	0.886

The Korsmeyer–Peppas model was applied only to the initial release phase (Mt/M∞ ≤ 0.60). The release exponent n was used as an indicative parameter of the release mechanism. The model was not applied where fewer than three valid early release points were available. N/A—not applicable.

## Data Availability

The raw data supporting the conclusions of this article will be made available by the authors on request.
